# Decaying *Raphia farinifera* Palm Trees Provide a Source of Sodium for Wild Chimpanzees in the Budongo Forest, Uganda

**DOI:** 10.1371/journal.pone.0006194

**Published:** 2009-07-10

**Authors:** Vernon Reynolds, Andrew W. Lloyd, Fred Babweteera, Christopher J. English

**Affiliations:** 1 Emeritus Professor, School of Anthropology, University of Oxford, Oxford, United Kingdom; 2 Faculty of Science and Engineering, University of Brighton, Brighton, United Kingdom; 3 Budongo Conservation Field Station, Masindi, Uganda and Faculty of Forestry and Nature Conservation, Makerere University, Kampala, Central Region, Uganda; Indiana University, United States of America

## Abstract

For some years, chimpanzees have been observed eating the pith of decaying palm trees of *Raphia farinifera* in the Budongo Forest, Uganda. The reasons for doing this have until now been unknown. An analysis of the pith for mineral content showed high levels of sodium to be present in the samples. By contrast, lower levels were found in bark of other tree species, and also in leaf and fruit samples eaten by chimpanzees. The differences between the *Raphia* samples and the non-*Raphia* samples were highly significant (p<0.001). It is concluded that *Raphia* provides a rich and possibly essential source of sodium for the Budongo chimpanzees. Comparison of a chewed sample (wadge) of *Raphia* pith with a sample from the tree showed a clear reduction in sodium content in the chewed sample. Black and white colobus monkeys in Budongo Forest also feed on the pith of *Raphia*. At present, the survival of *Raphia* palms in Budongo Forest is threatened by the use of this tree by local tobacco farmers.

## Introduction

**Figure 1:**
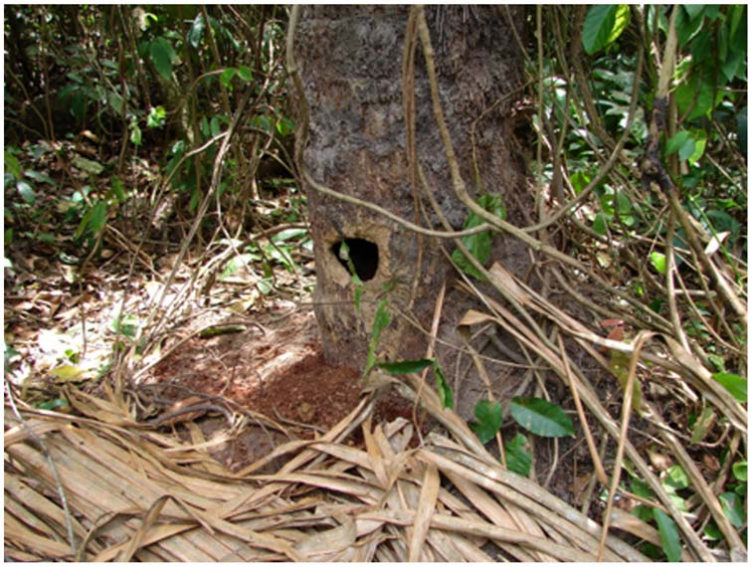
Decaying Raphia farinifera tree with medium sized hole.

The chimpanzees (*Pan troglodytes schweinfurthii*) of the Budongo Forest Reserve, Masindi District, Western Uganda, were initially studied by one of us (VR) in 1962 [1], and have been studied continuously since 1990 [2]. The Budongo Forest habitat consists of moist semi-deciduous tropical forest, and contains a number of forest types, notably *Cynometra* Forest, Mixed Forest, Colonising Forest, and Swamp Forest [3]. The study community of chimpanzees is named the Sonso community after the River Sonso which runs through its range. At various places along the river, Swamp Forest prevails. Swamp Forest contains several Raphia farinifera palm trees. After a single flowering and fruiting, the trees of this species lose their foliage and die. The dead bole remains standing and is 15–30 ft high. The bole rots down until it consists of a hard outer bark with a soft, moist, fibrous woody pith. Chimpanzees make a small hole in bark at the base of the dead tree with their teeth, widen it with their fingers and later their hands. Through the hole, they extract dead pith, chew it thoroughly, swallow the juice and some particles of woody matter, and finally spit out a fibrous wadge of chewed pith. There is frequently competition for *Raphia* pith. Eventually trees are abandoned, often with large holes and much inner pith removed (Fig. 1). Until now, the reason for consumption of the dead *Raphia* pith has not been known.

## Results

Table 1 shows the results of the chemical analysis expressed as mg per kg of dried material for each sample. In this table, results for elements for which no more than trace amounts were obtained have been omitted. With one exception (samples 7 and 8) samples were independent of each other, coming from different trees or at different collection dates. In the case of samples 7 and 8, sample 8 was a wadge (a spit-out chewed sample) of sample 7 and was omitted from the analysis. For purposes of analysis, the samples were placed into two groups: *Raphia* pith (n = 10), and all other samples (n = 16). Statistical tests were done using SPSS version 7. Data were not normally distributed (Lilliefors test p<0.000). Mean mineral contents of the two groups were therefore compared using non-parametric Mann-Whitney U-tests. Results are shown in Table 2.

**Table 1:** Results (mg kg^−1^).

Sample no.	Species*	Plant part	Magnesium	Sodium	Potassium	Calcium	Manganese	Iron	Zinc	Phosphorus	Sulfur
1	Rf	pith	293	7096	5118	815	60	90	31	58	16485
2	Rf	pith	621	3365	4892	1282	266	140	97	190	24472
3	Rf	pith	556	14616	6594	1332	139	23	144	293	19375
4	Rf	pith	440	1431	4518	421	366	67	71	88	6499
5	Rf	pith	5771	1666	12559	2756	1024	71	624	906	61301
6	Rf	pith	1118	5152	3165	679	254	37	164	204	47232
7	Rf	pith	6586	7194	11708	4568	1785	515	422	1057	127516
8**	Rf	pith	1449	1095	3915	1223	308	229	88	338	24984
9	Rf	pith	4560	8666	.	1829	232	20	147	261	21974
10	Rf	pith	2847	3216	7384	1327	145	159	115	437	31490
11	Rf	pith	2490	1920	.	962	97	60	41	205	13843
12	Fu	bark	265	66	3048	1521	20	12	13	59	5519
13	Fu	bark	340	75	2770	1452	28	14	11	73	6962
14	Cp	bark	443	800	2261	1005	11	301	−2	364	7886
15	Ab	bark	383	2455	.	1723	28	29	42	409	9368
16	Ab	bark	393	100	835	6912	11	37	37	533	7576
17	Me	bark	1203	198	1141	16183	106	57	7	213	15521
18	Zg	leaf	2740	289	.	2518	16	117	243	2273	95695
19	Fe	leaf	4114	408	24993	12449	81	118	69	1187	51291
20	Cm	leaf	3738	436	.	5790	253	86	68	1106	32835
21	Fv	leaf	1112	156	7479	3130	23	16	34	729	11478
22	Mi	fruit	1132	321	.	2007	44	21	32	815	16878
23	Bp	fruit	3634	546	.	10878	23	63	94	4381	32879
24	La	fruit	767	164	10073	2823	20	34	39	1359	15070
25	Fm	fruit	1751	135	.	4895	20	49	74	1145	26913
26	Fe	fruit	2889	192	.	7563	41	72	143	1962	41893
27	Be	fruit	1305	55	.	791	43	30	59	818	48638

* Rf = Raphia farinifera, Fu = Funtumia elastic, Cp = Cleistopholis patens, Abo = Astonia boonei, Zgo = Zanha golungensis, Fe = Ficus exasperate, Fv = Ficus varifolia, Fm = Ficus mucuso, Cm = Celtis mildbraedii, Mi = Mangifera indica, Bp = Broussonettia papyrifera, La = Lantana sp., Be = Beoquartiodendron oblanceolatum.

** wadge (see text).

**Table 2:** Mann-Whitney U-test results: raphia samples (without wadge sample) vs all other samples.

	magnesium	sodium	potassium	calcium	manganese	iron	zinc	phosphorus	sulphur
Mann-Whitney U	67.000	3.000	20.000	29.000	8.500	54.500	31.000	37.000	64.000
Wilcoxon W	203.000	139.000	56.000	97.000	84.500	190.500	167.000	92.000	200.000
Z	−.685	−4.058	−1.260	−2.813	−2.688	−1.344	−2.583	−2.266	−.843
Asymp. Sig. (2-tailed)	.493	.000	.208	.005	.007	.179	.008	.023	.399
Exact Sig. [2*(1-tailed Sig.)]	.517	.000	.234	.004	.006	.182	.007	.023	.421

**Figure 2:**
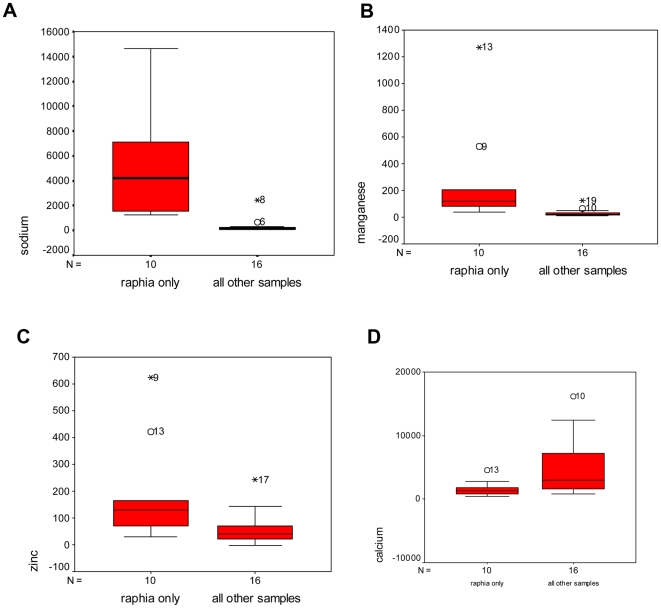
Box plots showing results for raphia samples (without wadge sample) vs all other samples: (a) sodium (b) manganese (c) zinc (d) calcium. All values are mg.kg^−1^.

As can be seen from Table 2, significant differences between group means (Raphia vs. all other samples) were found for four of the minerals tested, sodium, calcium, manganese and zinc. In the case of calcium (p = 0.004), higher values occurred in the ‘all other samples’ group and reflect the normal high level of calcium in leaves and fruits. In the case of sodium (p<0.000), manganese (p<0.006), and zinc (p = 0.007) higher values occurred in the *Raphia* group. Bark, leaves and fruits from other tree species did not contain the high levels of sodium found in the *Raphia* samples. These results are illustrated by box-plots (Fig. 2).

## Discussion

Since study of the Sonso community of chimpanzees began in 1990, individuals and small parties of the animals have been seen occasionally eating the pith of dead Raphia trees, but until now the reason for doing so has not been known. The first report showing high levels of sodium in samples of dead wood eaten by apes was that of Rothman et al for mountain gorillas (*Gorilla gorilla beringei*) in Bwindi Impenetrable National Park, Uganda [4]. In that study it was estimated that the sodium obtained from dead wood constituted 95% of the sodium in the gorillas' diet. Using similar methods to those employed here, a mean dry sodium weight of 810.7 mg kg^−1^, with a range of 100–1920 mg kg^−1^ was measured.. This compares with the mean dry sodium weight for Raphia samples in the present study of 5432 mg kg^−1^, range 1431–14616 mg kg^−1^. Raphia palm trees in Budongo Forest therefore offer higher concentrations of sodium to the chimpanzees than dead wood does to the Bwindi gorillas.

Rothman et al found that sodium levels were significantly higher in dead wood than in other items in the diet [4], and this was also the case in the present study. As they point out, sodium is an essential item of diet for apes, lack of which has wide-reaching effects on health [5]. In the Budongo Forest too, there appears to be little sodium in the other plant parts eaten and tested here (bark, leaves and fruits), although sodium is present in small quantities. In particular, the greater part of the chimpanzees' daily diet (up to 95%) consists of leaves and fruits [6] which contain only small quantities of sodium. There is however one important dietary difference between chimpanzees and mountain gorillas. The Budongo chimpanzees eat meat sporadically, mainly in the form of colobus monkeys (*Colobus guereza*) which are hunted, and this provides an additional source of sodium. The interaction between Raphia eating and meat eating was not explored in this study.

As in the present study, Rothman et al found significantly lower values for calcium and phosphorus in decaying wood than in the rest of the diet [4]. They also found lower values for manganese which was not the case in the present study, in which manganese was higher in the Raphia samples (p = 0.059). The reason for this may be the high levels of manganese in groundwater along the Albertine Rift [7]. Mahaney et al found high levels of manganese in clay eaten by chimpanzees living in the Mahale mountains, Tanzania, also along the Rift Valley [8]. Significantly lower values for other minerals, magnesium, potassium, zinc, and copper found by Rothman et al were not found in the present study [4].

Black and white colobus monkeys (*Colobus guereza*) are also known to consume *Raphia* pith in Budongo Forest (pers. comm., field assistants at BCFS) and during the present study hairs of this species were twice found at *Raphia* feeding sites. Oates found high levels of sodium, iron, manganese and zinc in swamp plants eaten by black and white colobus monkeys living in Kibale Forest, western Uganda, also along the Albertine Rift [9]. High mineral content was also found in some clays collected from stream-banks in the forest. Concentrations for swamp plants and clays were higher than in dry-land leaves, buds and fruits constituting the major part of the monkeys' diet.

An interesting feature of the samples collected concerns samples 7 and 8. These were the only two non-independent samples collected, coming from the same tree during the same observation period. Sample 7 consisted of Raphia pith collected with a knife from inside the tree. Sample 8 consisted of a discarded wadge of Raphia pith from the same tree, collected on the ground at the feeding site. Comparison of the sodium content of these two samples shows that the uneaten Raphia pith (sample 7) contained 7194 mg kg^−1^, whereas the chewed and spat-out wadge (sample 8) contained 1095 mg kg^−1^, evidence that the individual which chewed this sample did indeed ingest sodium.

In recent years *Raphia farinifera*, a tree we now know to provide the chimpanzees of Budongo Forest with essential sodium, has become scarcer. Besides natural predators such as baboons and pigs that eat its shoots, it provides humans with two products. The living trees are occasionally felled and the trunk opened to allow air into the pithy centre, which then ferments and produces an alcoholic palm wine beverage which is bottled and sold. This, however, is not thought to be the main cause of the decline in numbers of *Raphia*. The main danger to this species comes from local tobacco farmers, who kill the tree during its growth period, before flowering and fruiting, in order to strip its leaves for *Raphia* string (‘raffia’), which is then used to tie tobacco leaves during and after the the drying and curing process [10]. As a result, *Raphia* palms are becoming scarce in Budongo Forest. Tobacco farmers and British American Tobacco, a company which buys much of the crop, will be approached about this problem and it is hoped a solution may be found.

## Materials and Methods

### (a) Field collection

Between 16 Feb and 2 April 2008, samples of pith (including one wadge) from *Raphia* trees were collected, in each case when chimpanzees had been observed feeding on the trees just before collection. Samples weighed <50 g. Pith samples were obtained from the inside of trees with use of a knife. The wadge was collected from where it was dropped, beside the hole in the tree. Each sample was placed into a sample tube using gloves or tweezers, tubes were closed and marked with sample no., date, tree location, species of tree, name(s) of chimpanzees feeding on the tree, nature of the sample (bark, pith or wadge), initials of collector. Collectors were in all cases trained field assistants of BCFS or in one case VR. During the same time period, samples of rotten wood, bark, leaves and fruits seen being eaten by chimpanzees were collected in the same way, in adjacent forest, to be analysed for comparison with the *Raphia* samples.

All samples were taken within a period of 2 hours to the field base where they were removed from the bags with tweezers and dried separately in a warm dry space at room temperature. Samples were dry in 1–3 days and all dried satisfactorily. The dry samples were placed in polythene bags which were sealed, marked, and taken by air to the UK for analysis. In the UK prior to analysis they were kept in a dry room at room temperature. No samples were spoiled during the pre-analysis period.

### (b) Laboratory analysis

The samples of material were dried to constant weight in an oven at 105°C. The samples were then ashed at 550°C in a pyrolysis oven. The total mass of the ashed material was determined before digesting a sample (circa 0.1 g) of each material in 3 mL of aqua regia in a water bath at 100°C for 2 hours. The digested samples were diluted to 10 mL using distilled water before serially diluting to obtain 1 in 10, 1 in 100 and 1 in 500 dilutions. The elemental content of each sample was then determined using a Perkin Elmer Optima 2100 DV Inductively Coupled Plasma Optical Emission Spectrometer (ICP-OES) using 5 and 10 mg L^−1^ elemental standards.

### Further Information

Still images and video clips of Raphia eating are available from the first author and from C. Hobaiter, School of Psychology, St Andrews University, email: clh42@st-andrews.ac.uk
